# Optimal sizing and operation of a hybrid energy systems via response surface methodology (RSM)

**DOI:** 10.1038/s41598-024-71035-9

**Published:** 2024-08-30

**Authors:** Arash Moradzadeh, Kazem Pourhossein, Amin Ghorbanzadeh, Morteza Nazari-Heris, Ilhami Colak, S. M. Muyeen

**Affiliations:** 1 Department of Electrical Engineering, Qatar University, Doha, Qatar; 2 Faculty of Electrical and Computer Engineering, University of Tabriz, Tabriz, 51666 Iran; 3 Department of Electrical Engineering, Tabriz Branch, Islamic Azad University, Tabriz, Iran; 4 Department of Engineering, East Carolina University, Greenville, NC USA; 5 Faculty of Engineering and Architecture, Department of Electrical and Electronics Engineering, Nisantasi University, Istanbul, Turkey

**Keywords:** Energy infrastructure, Energy storage, Renewable energy

## Abstract

Hybrid energy systems (HESs) are the most important sources of energy demand-supply, have developed significantly around the world. Microgrids, renewable energy sources, remote telecommunications stations, greenhouses, etc., are being considered as HESs applications. Optimal sizing of these systems is considered as one of the important issues related to energy management. In this paper, the Response Surface Methodology (RSM) is proposed for the optimal sizing of a Photovoltaic (PV) system in a HESs. The suggested procedure solves the optimization problem by considering the factors affecting PV output power about the environmental conditions of the HESs. Providing a mathematical model for each of the input parameters and the ability to assessment the sensitivity of each of the input variables are the most important advantages of the proposed technique. In this paper, the RSM provides the most optimal sizing related to the PV system by considering climatic and geographical factors in the study site, and technical and economic issues related to the HESs. The optimal model obtained is evaluated by the Analysis of Variance (ANOVA) evaluation method, which is one of the important techniques of statistical evaluation. It should be noted that the RSM technique can be utilized to optimize all components of any HES.

## Introduction

Sustainable and reliable energy generation suffers from drawbacks with various aspects. Including its reliance on fossil fuels, which on the one hand increased greenhouse gases and is depleting worldwide. This dependency, in addition to environmental contamination, will increase electricity prices, imbalances between supply and demand of energy, and as well as reduce the reliability of the power and energy systems^1^. In contrast, HESs are considered as an electric energy system which is consisting of several renewable and un-renewable energy sources. These energy systems can operate in two off-grid (standalone) or grid connected modes. Increasing power system reliability, reducing environmental pollution, and eliminating economic limitations are prominent features of the HESs. Microgrids, Greenhouses, Remote Telecommunications Stations, Off-Grid Buildings, Renewable Energy Sources (RESs), Water Pumping Systems, Marine and Offshore Platforms, Military Installations, etc., are being considered as applications of HESs^2^. In the meantime, the RESs have been able to expand dramatically around the world based on prominent concepts such as being an accessible, low cost, and environmentally friendly. The RESs are used to meet approximately 15% of the energy demands. Bioenergy, hydropower, ocean energy, hydrogen and fuel cells, solar energy, and wind energy are the most important RESs^3,4^. These energy sources are called alternative energy sources and they are clean, cheap, stable, and accessible. Natural gas, oil, and coal are considered as the main types of fossil fuels in which there are some problems. Fossil fuels are expensive and limited, and in addition have environmental issues, such as increased CO_2_ gas emissions^5,6^. RESs are going to play a pivotal role in the electricity environment future in which they were divided into three categories as fossil fuels, RESs, nuclear sources. Due to high power transferring costs, photovoltaic (PV) panels are one of the suitable options in rural places and that is why the use of the RESs is so important^7^. These systems are less complex and have lower losses. In many cases, renewable systems are integrated with fossil fuel power plants which in this case, cogeneration increases the operation of the system. For example, the optimal performance of a system consisting of the PV, fuel, and combustion engine is presented in Refs.^8,9^. In these studies, minimizing the overall cost is the objective of optimization procedure and the multi-objective optimization is performed by Pareto optimal set. Optimal sizing associated with a HES consisting of wind and hydro turbines in an island system is presented in Ref.^10^. This study determines the payback period and the impact of renewable resources. To increase the resilience of the system and face the uncertainty of the PV output, different types of storage systems, diesel generators and, the combustion engine can be used^11^. Economic factors are the alternatives in which they must be considered in the optimization of the model. The economic factors can be divided into three general categories: total operation cost of the system, the capital cost that depends on the environmental and economic factors, and finally the current value of the system^12,13^. Various sizing methods have been proposed in recent years. In Graphic construction methods average of the wind speed and solar radiation are considered hourly or monthly and some systems such as PV-battery and PV-wind turbines are considered as energy sources. In these systems, the slope angle of the PV system and the installation height of the wind turbine are considered as the limitation of this method^14^. This method is used to calculate the optimal size of the battery and the PV system in a hybrid PV/wind system. Wind speed and solar radiation data have been collected daily for 30 years. Then, with this daily data, the amount of output power generated by PV panels and wind turbines is calculated hourly during a day. In other studies^15,16^, the probabilistic method is introduced for improving the optimal sizing issues. In a probabilistic method, sizing of the PV panels and wind turbines are considered as input, and the solar, wind, and battery storage systems are selected as energy sources. The dynamic performance related to the hybrid system is not illustrated in the Probabilistic method^16^. In 2013, a fast response-based probabilistic method has been used in a valuable study^17^. The fast response method is based on measuring fast response reserves based on the output fluctuations distribution in a settlement interval^17^. In other valuable studies, a hybrid PV/wind system has been suggested as an independent system^8,9^. To determine the amount of production power and storage, a residential area has been studied. These production and storage units are designed to supply the annual load and minimize overall costs^8^. In iterative methods, the average of the wind speed and the solar radiation is considered in this method, or sizing of the PV panels and speed of the wind turbines are considered as inputs in this method. The slope angle of the PV system and the installation height of the wind turbine are considered as the limitation of this method. Linear changing of the decision variables causes to reach suboptimal solutions^10,18,19^. In the selection of artificial intelligence and hybrid techniques^20–22^, the average of the wind speed and solar radiation as well as sizing the wind turbine and PV systems are considered as input parameters. Low flexibility in the designing of the system is considered as the limitation of this approach. In Ref.^23^, the genetic algorithm optimization method has been used for optimal system design and location. In genetic algorithms, the studied systems are compared with real systems. The basic objective of the genetic algorithm is to achieve a universal optimization method. In Ref.^21^, the Biogeography Based Optimization (BBO) algorithm has been employed to obtain the optimal size of system components and minimize costs in a remote area in India. The proposed system uses a diesel generator to ensure alternative power generation. The BBO method has a very high degree of convergence, short computation time, and achieves good convergence in the fastest time, and offers a suitable solution^21^. In another study, the support vector machine (SVM) network is selected for sizing optimization^22^. A comprehensive evaluation based on the optimal sizing of a HES including PV/Pump-hydro storage (PHS), Diesel/PHS and PV/Diesel/Battery has been performed in Ref.^24^ via hybrid optimization associated with multiple energy resources software. In Ref.^25^, a novel optimization approach based on integrating a biomass system with a PV, wind turbine, and battery system has been suggested to increasing power supply and minimizing energy costs in rural regions. Table 1 lists the types of sizing methods and compares them technically.

**Table 1:** Different types of sizing methods.

Methods	Description	Disadvantages	Refs
Graphic construction methods	Average wind speed and solar radiation are selected in this method Some systems such as PV-battery and PV-wind turbines are considered as energy sources	The slope angle of the PV system and the installation height of the wind turbine are considered as the limitation of this method	^14^
Probabilistic method	Sizing of the PV panels and wind turbines are considered as input while solar, wind, and batteries storage systems are considered as energy sources	The dynamic performance related to the hybrid system is not illustrated in this technique	^15,16^
Analytical methods	Average wind speed and solar radiation are selected in this method Considered solar irradiation, wind speed, battery storage systems, biomass systems are depending on the type of software	Low flexibility in the designing process can be considered a limitation	^8,9,26^
Iterative methods	Average of the wind speed and the solar radiation is considered in this method or sizing of the PV panels and, wind turbines are considered as input in this procedure	The slope angle of the PV system and the installation height of the wind turbine are considered as the limitation of this method	^10,18,19^
Artificial intelligence and hybrid methods	Average of the wind speed and solar radiation as well as sizing the Wind turbine and PV systems is considered in this method	Low flexibility in the designing of the system is considered as the limitation of this approach	^22,27^

According to the reviewed methods, the mentioned models are so complicated. Also, factors such as the environmental factors and consumers' preferences are not considered. Various types of approaches that are employed to optimize the HESs are reviewed and listed in Table 2.

**Table 2:** Different types of algorithms to optimize the hybrid systems.

Methods	Description	Advantages	Disadvantages	Refs
Genetic algorithm (GA)	It is operated based on natural evolution	Provide several solutions for the optimized model. Also, a specific toolbox is provided for this approach in the MATLAB software	The speed of the calculation and convergence is lower than other approaches	^23^
Particle swarm optimization (PSO)	It is operated based on bird and fish movement	The searching speed is so high Also, the instructor of this approach is so simple in comparison to other plans	Optimizing the non-coordinated system is so hard with the use of this approach	^28–30^
Simulated annealing	It is operated based on the annealing process	Nonlinear and chaotic models can be solved easily with the use of this approach Furthermore, reaching global optimality is easy, too	Appropriate setting the different classes of the constraints is so crucial to obtain the optimal solution	^31^
Ant algorithms	It is operated based on the ants’ behavior	It is a suitable approach to find local and global solutions under several optimization problems	The number of controlling parameters is so high in this approach in which all of them must be tuned	^32^
Bee-inspired algorithms	It is operated based on the bees’ behavior	It is a suitable approach to find local and global solutions under several optimization problems. It can easily combine with other optimization approaches	The number of controlling parameters is so high in this approach	^33,34^
Harmony search	It is operated based on jazz music	This approach can optimize the discontinuous function and discrete variables without any difficulties Setting the initial values is not required in this approach	The process of solving is so complicated	^29–31,35^
Biogeography-based optimization (BBO)	It is operated based on the immigration process of the animals	The computation time is so high Converging the results is the other strong point of this approach	This approach is weak in finding the globally optimal result There is no provision to find the best member of the assumed generations	^34,36^
Gravitational search algorithm	It is operated based on Newton’s attraction law	High speed in converging the results is one of the strengths of this approach High accuracy in the calculation is the other point in this approach	The premature convergence process is so complicated in this approach	^37^
Imperialist competition algorithm	It is based on social and political movements	The convergence accuracy is so high in this approach. This approach can easily handle the high dimension of nonlinear hybrid systems	The process of computation is so complicated	^38,39^
Hybrid optimization techniques	It is operated based on the integration of several algorithms	The accuracy of the obtained results is high. Also, the computational time is low in the mentioned approach	The process of writing the code is so complicated	^40–42^

In this paper, a novel technique called Response Surface Methodology (RSM) has been proposed for the optimization of a PV system in a HES. This hybrid system is responsible for providing the amount of electricity power, heat, and water demand by an area. The studied system comprises the PV, battery storage internal combustion engine, boiler, fuel tank, and water storage. The PV and internal combustion engines are utilized to generate heat and electricity. Also, fuel is utilized in the boiler and internal combustion engine to generate heat. Then, a pump is used to produce hot and cold airflow and storage sources for hot and cold air and water are considered. In this method, simultaneous size and performance optimization is performed, so that the amount of consumption and conditions of the area during 24 h are considered. In the proposed system, electricity, heat, hot and cold air, and water are provided and various parts of the system are optimized. In this study, for different parts of the system, an equation appropriate to the conditions of the region is presented. Then, the RSM technique is selected to determine the optimal size of components such as PV panels, inverter, etc., and reduce the computational burden. Selecting the value of variables with a minor amount of error is one of the most important advantages of the proposed procedure. In addition, the RSM can model the performance of all system components individually in mathematical formula mode. Thus, the performance of each component in the whole system can be easily analyzed. To express the effectiveness of the proposed model of this study, a comparative analysis between proposed RSM-based approach and several well-known optimization techniques, including GA, PSO, and BBO is performed. This comparison highlights:

- *Computational Efficiency:* The RSM demonstrated superior computational efficiency, particularly in scenarios with complex, multi-modal landscapes, due to its capability to reduce the number of simulations required.
- *Solution Quality:* Suggested RSM method produced competitive or superior solutions in terms of the objective functions, especially in balancing multiple criteria such as cost and energy efficiency.
- *Flexibility and Scalability:* The RSM-based approach provided flexibility in handling various system constraints and scales, outperforming traditional methods in adaptability.

To clarify, the novelty of this research lies in the following aspects:

Integration of RSM in HES optimization:

While Response Surface Methodology (RSM) has been widely used in various fields, its application in optimizing HES, particularly for the simultaneous sizing and operation strategy, remains limited. This study pioneers this integration, offering a systematic and computationally efficient approach to address the complexity of such systems.

Comprehensive system design:

Proposed method uniquely combines RSM with multi-objective optimization, accounting for both technical and economic factors. This dual focus ensures that the designed system is not only optimized for performance but also for cost-effectiveness, which is crucial for practical implementation and scalability.

Case study and validation:

This paper has conducted a detailed case study that demonstrates the practical applicability and advantages of suggested method in real-world scenarios. This empirical validation helps to illustrate how our approach can lead to more efficient and cost-effective energy solutions compared to traditional methods.

The organization this paper in the rest is as follows: Section “Case study” introduces the case studied. The problem formulation is described in Section “Problem formulation”. The application of the proposed procedure and design variables is described in Sect. “Design variables”. The results are presented in Sect. “Results”. Finally, Sect. “Conclusion” concludes the paper.

## Case study

**Fig. 1:**
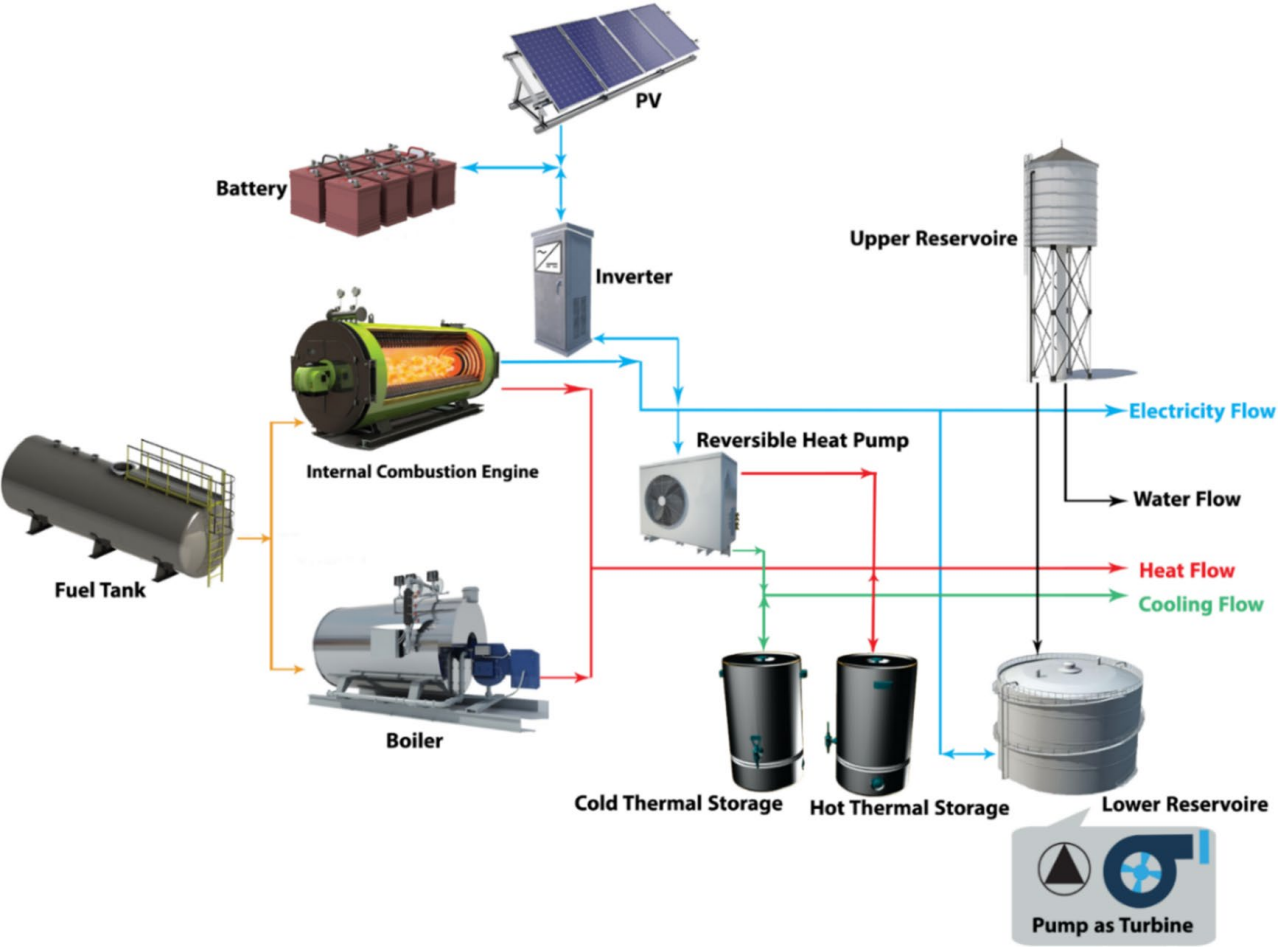
The structure of the system.

The studied test system is a PV-based combined cooling, heat and power (CCHP) system^43^ that is located in North West of Iran. The solar irradiation of the region is between 1700 and 1800 kWh/m^2^. The studied system is comprised of a PV panel, internal combustion engine, boiler, reversible heat pump, pump as turbine, electrical energy storage (battery), inverter as power transformation which converts the DC output of the battery to the AC and thermal and cooling energy storage. Thermal energy can be produced by the combustion engine, boiler heat losses, and by the heat pump. Also, the heat pump can produce cooling energy by working in reverse mode. The fuel tank is used as a fuel of the system and, upper and lower reservoirs are utilized to store the water. Table 3 shows the required amount of water, electricity, and cooling and heating energy for a day. During the winter days, the required amount related to heating energy and electricity increases, and the amount of cooling energy has reached 0 kWh. The plant schematic is shown in Fig. 1. According to this figure, the proposed system is a hybrid system that can supply electricity and cooling, and heating, which are called trigeneration systems. Trigeneration systems are up to 50% more efficient annually than power plants of the same size. This plant is consists of a PV system, an internal combustion engine (ICE), a boiler (BL), and two pumps, one as a reversible heat pump (RHP) and one as a pump as a turbine (PAT). The PV and ICE systems are used to generate electricity. The fuel Tank plays the role of fueling the BL and ICE. BL and ICE are used to generate heating and cooling energy. By using the RHP, the cooling energy requirement can be met in reverse mode. In the introduced system, battery (BAT), cold thermal storage (CS), hold thermal storage (HS), upper reservoir (UR), and a lower reservoir (LR) are storage systems. The amount of radiation (G), temperature (T), cost (C) and longitude (E), and height of upper reservoir (H) are considered as effective parameters on this system. These data are considered appropriate to the study area. Water is moved between two sources by the PAT. The height difference between the two reservoirs is considered to be 50 m.

**Table 3:** The demands in the studied system.

The need for water and energy In a day of winter		The need for water and energy In a day of Summer	
Demand	Value	Demand	Value
Electrical power	742 kWh	Electrical power	699 kWh
Thermal energy	6192 kWh	Thermal energy	1161 kWh
Cold Energy	0 kWh	Cold Energy	3505 kWh
Water demand	17.6 ${\text{m}}^{3}$	Water demand	19.4 ${\text{m}}^{3}$

## Problem formulation

The electrical and thermal balance is provided in Eqs. (1) and (2)^44^.

1 $$If ({P}_{PV}+ {P}_{BAT})> 0\text{ then }{P}_{us}= \left({P}_{PV}+ {P}_{BAT}\right)*{\eta }_{INV }+ \left({P}_{ICE}+ {P}_{PAT}\right)$$

2 $$Otherwise\, {P}_{us}= \frac{\left({P}_{PV}+ {P}_{BAT}\right)}{{ \eta }_{INV }}+{P}_{ICE}+ {P}_{PAT}$$

3 $${Q}_{h,us}= \left({Q}_{ICE}+ {Q}_{Bl}\right)+ {Q}_{h,RHP}+ {Q}_{HS}$$

4 $${Q}_{c,us}={Q}_{c,RHP}+ {Q}_{CS}$$

where ${P}_{PV}$ is the generated electric power by PV and ${P}_{BAT}$ is the storage power in BAT. ${P}_{us}$ is electricity demand. ${P}_{ICE}$ shows the power produced by ICE, ${P}_{PAT}$ is the power of PAT, and ${\eta }_{INV}$ represent the inverter coefficient. If the sum of ${P}_{PV}$ and ${P}_{BAT}$ is greater than 0, the amount of user power can be calculated by Eq. (1) otherwise, it can be calculated from Eq. (2). ${Q}_{ICE}$ demonstrate the heat generated by ICE and ${Q}_{Bl}$ illustrate the heat generated by BL. ${Q}_{h,RHP}$ and ${Q}_{c,RHP}$ are the heat and cooling generated by the reversible heat pump, respectively. ${Q}_{HS}$ and ${Q}_{CS}$ are the heat and cool that stored in HS and CS. ${Q}_{h,us}$ and ${Q}_{c,us}$ depicts the heat and cool demand, respectively.

The water flow rates balance is considered other constraint:

5 $${m}_{UR}={m}_{us}+ {m}_{PAT}$$

where ${m}_{UR}$ represents the flow rate of the tank. The variable ${m}_{us}$ denotes the water demand of the resort, and ${m}_{PAT}$ corresponds to the flow rate of the pump. The maximum and minimum flow rates of the $PAT$, as well as the maximum and minimum State of Charge (SOC) of the batteries, along with the maximum charging/discharging rates, and the maximum and minimum loads for the $ICE, \,RHP, \,and\, BL$, are established based on the devices' specifications. To prevent disruptions in system management for the subsequent days, it is required that, by the end of each day, the water level in the tank, the $SOC$ of the batteries, and the state of the thermal storage systems be restored to their initial conditions as observed at the beginning of the day^44^:

6 $${V}_{UR,h=24}={V}_{UR,h=0}$$

7 $${SOC}_{h=24}={SOC}_{h=0}$$

8 $${TH}_{HS,h=24}={TH}_{HS,h=0}$$

9 $${TH}_{CS,h=24}={TH}_{CS,h=0}$$

The optimization problem is formulated as a single objective function that incorporates the costs associated with the devices, fuel consumption, and penalties for any constraint violations. The final equation is:

10 $$F\left({X}_{j}\right)=min\left[f\left({X}_{j}\right)+\sum_{z=1}^{nc}{\lambda }_{z}{\left[{VIOL}_{z}\right]}^{2}\right]$$

where $F\left({X}_{j}\right)$ represents the cost function, ${\lambda }_{z}$ is the penalty multiplier, and ${VIOL}_{z}$ denotes the magnitude of the violation for constraint $z$.

Finally, the objective function of the studied system is provided as follows^44^:

11 $${F}_{cost}= {C}_{PV}{S}_{PV}+{C}_{BAT}{S}_{BAT}+{C}_{INV}{S}_{INV}+{C}_{ICE}{S}_{ICE}+{C}_{BL}{S}_{BL}+{C}_{RHP}{S}_{RHP}+{C}_{PAT}{S}_{PAT}+{C}_{UR}{S}_{UR}+{C}_{HS}{S}_{HS}+{C}_{CS}{S}_{CS}+ \sum_{h=1}^{24}({C}_{f}{m}_{ f.h}) \Delta t$$

where $C$ shows the cost of each component, *S* represents the size of each component, ${C}_{f}$ is the fossil fuel cost, and ${m}_{f.h}$ demonstrate the total amount of fuel consumed by the BL and the ICE. Table 4 shows the estimated costs for each system component and fuel.

**Table 4:** Costs of all components.

PV	ICE	BAT	INV	PAT	TCH	RHP	UR	BL	HS	CS	Fuel
340€	1000€	210€	500€	220€	200€	300€	100€	51€	38€	20€	1.4€

**Fig. 2:**
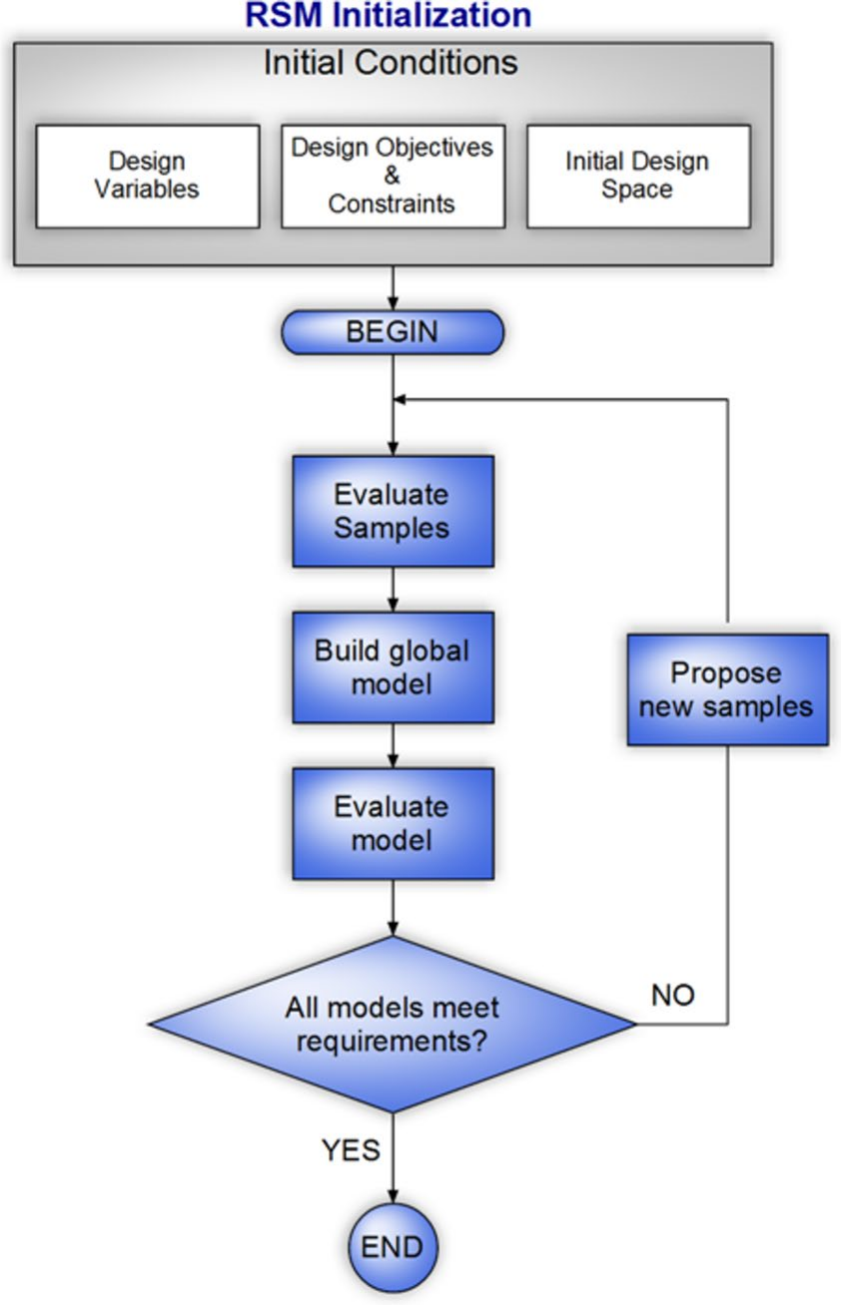
The RSM flowchart.

The Design of Experiments (DOE), which is a quality improvement method allows to users for determining the sensitivity of each component to the various variables in the studied test system. The output of this method is a mathematical formulation that is determined based on the nature of the operation. It is noteworthy that the mentioned formulation is so exact and stable. Also, this constructed formulation can help us to determine the size of the components without any difficulties. Finally, the environmental and climate conditions, which are essential factors in sensitivity calculation are considered in the mentioned approach while it is not found in the PSO approach^44^. Fill factorial, RSM, mixture, and Taguchi are various types of DOE applications which the RSM method is selected to use in this paper. The low computation burden of the RSM is the significant merit of this approach to the PSO. In other words, this method is provided a new formulation for each component of the studied system instead of giving a final strategy that is calculated based on the reputation concept^45^. As shown in Fig. 2 which is the RSM flowchart, the input data of the RSM are the meteorological parameters and the cost of system components. In this study, G, T, C, E, H are the meteorological parameters and design variables. In this state, should define the minimum and maximum values of inputs. Then, the full factorial design is selected to calculate the model and the evaluating the impact of parameters (sensitivity analysis). In the following the size of each component is defined as new parameters. The parameters range was defined by minimum and maximum levels as shown in Table 5. In the next step, the RSM evaluates samples and makes a global model.

**Table 5:** Parameters and levels.

Parameters	${S}_{PV}$	${S}_{BAT}$	${S}_{INV}$	${S}_{ICE}$	${S}_{BL}$	${S}_{RHP}$	${S}_{PAT}$	${S}_{UR}$	${S}_{HS}$	${S}_{CS}$
Minimum level	600	400	70	80	80	146	5	90	500	1220
Maximum level	800	500	80	200	100	161	7	120	600	1500

## Design variables

The Central composite design toolbox in the MINITAB software has been employed to solve the aforementioned problem. In this regard, the minimum and maximum values related to the variables are entered as input data in a simulation. The obtained results for different components are reported in Tables 6. Also, Table 7 is the other outputs of the simulation that are calculated based on the various environmental conditions. Table 6 shows, the series of the RSM for each component, 31 experiments in the optimization procedure. Table 7 shows, the series of the Response Surface Methodology for environmental conditions, it includes 30 experiments for different combinations of the input variables.

**Table 6:** Complete response surface methodology column of components size design.

StdOrder	RunOrder	PtType	Blocks	Spv	Sice	Sbat	Sinv	Spat	Srhp	Sur	Sbl	Shs	Scs
36	1	1	1	800	200	400	70	5	161	90	100	500	1500
54	2	1	1	800	80	500	70	7	161	90	100	600	1220
134	3	− 1	1	700	140	500	80	6	153.5	105	90	550	1360
106	4	1	1	800	80	400	90	5	161	120	100	500	1220
113	5	1	1	600	80	400	70	7	161	120	80	500	1220
15	6	1	1	600	200	500	90	5	146	90	100	500	1500
102	7	1	1	800	80	500	70	5	161	120	80	500	1220
119	8	1	1	600	200	500	70	7	161	120	80	500	1500
1	9	1	1	600	80	400	70	5	146	90	100	600	1500
147	10	− 1	1	700	140	450	80	6	153.5	105	90	550	1220
140	11	− 1	1	700	140	450	80	6	160	105	90	550	1360
62	12	1	1	800	80	500	90	7	161	90	100	500	1500
18	13	1	1	800	80	400	70	7	146	90	80	500	1220
37	14	1	1	600	80	500	70	5	161	90	80	500	1500
98	15	1	1	800	80	400	70	5	161	120	100	600	1500
69	16	1	1	600	80	500	70	5	146	120	100	500	1220
8	17	1	1	800	200	500	70	5	146	90	80	600	1500
74	18	1	1	800	80	400	90	5	146	120	100	500	1500
50	19	1	1	800	80	400	70	7	161	90	80	500	1500
16	20	1	1	800	200	500	90	5	146	90	80	500	1220
100	21	1	1	800	200	400	70	5	161	120	80	500	1500
126	22	1	1	800	80	500	90	7	161	120	80	500	1500
70	23	1	1	800	80	500	70	5	146	120	80	500	1500
136	24	− 1	1	700	140	450	90	6	153.5	105	90	550	1360
55	25	1	1	600	200	500	70	7	161	90	100	500	1500
148	26	− 1	1	700	140	450	80	6	153.5	105	90	550	1500
5	27	1	1	600	80	500	70	5	146	90	80	500	1220
103	28	1	1	600	200	500	70	5	161	120	80	600	1500
43	29	1	1	600	200	400	90	5	161	90	80	600	1500
30	30	1	1	800	80	500	90	7	146	90	100	500	1220
57	31	1	1	600	80	400	90	7	161	90	100	600	1500

**Table 7:** Response surface methodology column for environmental condition.

StdOrder	RunOrder	PtType	Blocks	G	T	C	E
17	1	− 1	1	1650	12.5	340	38.5
19	2	− 1	1	1750	− 24.5	340	38.5
10	3	1	1	1800	− 6	339	39
20	4	− 1	1	1750	49.5	340	38.5
4	5	1	1	1800	31	339	38
16	6	1	1	1800	31	341	39
24	7	− 1	1	1750	12.5	340	39.5
8	8	1	1	1800	31	341	38
15	9	1	1	1700	31	341	39
11	10	1	1	1700	31	339	39
5	11	1	1	1700	− 6	341	38
22	12	− 1	1	1750	12.5	342	38.5
12	13	1	1	1800	31	339	39
23	14	− 1	1	1750	12.5	340	37.5
21	15	− 1	1	1750	12.5	338	38.5
30	16	0	1	1750	12.5	340	38.5
7	17	1	1	1700	31	341	38
1	18	1	1	1700	− 6	339	38
13	19	1	1	1700	− 6	341	39
9	20	1	1	1700	− 6	339	39
2	21	1	1	1800	− 6	339	38
26	22	0	1	1750	12.5	340	38.5
3	23	1	1	1700	31	339	38
28	24	0	1	1750	12.5	340	38.5
31	25	0	1	1750	12.5	340	38.5
6	26	1	1	1800	-6	341	38
27	27	0	1	1750	12.5	340	38.5
25	28	0	1	1750	12.5	340	38.5
14	29	1	1	1800	− 6	341	39
29	30	0	1	1750	12.5	340	38.5

Standard order (StdOrder) is the non-randomized order of the runs while run order (RunOrder) is a randomized order of the terms. Point type (PtTyoe) contains 3 levels that are 0, -1 and 1. 0 indicates the center point, 1 is a corner point, and -1 is an axial point.

Variance Inflation Factor (VIF) indicated the correlated status of the parameter. In other words, VIF = 1 indicted that the data do not have any correlation with one another, and 1 < VIF < 5 indicates the moderate relationship between the parameters and finally 5 < VIF < 10 suggests that the settings have a high association with each other.

The Standard Error (SE) coefficient is used to avoid the repeated results in the selection process. The T-Value is responsible for calculating the ratio between the factor and the standard error of each parameter.

P-Value is considered as a probability that is used to measure the evidence against the null hypothesis. By reducing the amount of expectations, stronger evidence is obtained against the null hypothesis.

In the second step, to determine the final problem formulations, the obtained results of Tables 6 and 7 are mixed with each other. For instance, Table 8 indicates the mixture of the ${S}_{PV}$ that is reported in Tables 6 and 7. Also, the series of the Response Surface Methodology for ${\text{S}}_{\text{PV}}$, it includes 31 experiments for different combinations of the environmental variables which has affected the size are shows in Table 8. The ANOVA table for the coded coefficients in the studied system model is presented in Table 9.

**Table 8:** Response surface methodology column for $Spv$.

StdOrder	RunOrder	PtType	Blocks	G	T	C	E	Spv
12	1	1	1	1800	31	339	39	800
15	2	1	1	1700	31	341	39	800
3	3	1	1	1700	31	339	38	700
22	4	− 1	1	1750	12.5	341	38.5	800
26	5	0	1	1750	12.5	340	38.5	600
4	6	1	1	1800	31	339	38	600
30	7	0	1	1750	12.5	340	38.5	800
17	8	− 1	1	1700	12.5	340	38.5	600
1	9	1	1	1700	− 6	339	38	600
24	10	− 1	1	1750	12.5	340	39	700
18	11	− 1	1	1800	12.5	340	38.5	700
6	12	1	1	1800	− 6	341	38	800
11	13	1	1	1700	31	339	39	800
13	14	1	1	1700	− 6	341	39	600
16	15	1	1	1800	31	341	39	800
2	16	1	1	1800	− 6	339	38	600
8	17	1	1	1800	31	341	38	800
20	18	− 1	1	1750	30	340	38.5	800
7	19	1	1	1700	31	341	38	800
27	20	0	1	1750	12.5	340	38.5	800
19	21	− 1	1	1750	− 6	340	38.5	800
14	22	1	1	1800	− 6	341	39	800
10	23	1	1	1800	− 6	339	39	800
21	24	− 1	1	1750	12.5	339	38.5	700
25	25	0	1	1750	12.5	340	38.5	600
9	26	1	1	1700	-6	339	39	700
31	27	0	1	1750	12.5	340	38.5	600
29	28	0	1	1750	12.5	340	38.5	600
5	29	1	1	1700	− 6	341	38	600
28	30	0	1	1750	12.5	340	38.5	800
23	31	− 1	1	1750	12.5	340	38	600

**Table 9:** The ANOVA table for the coded coefficients in the studied system model.

Coded coefficients					
Term	Coef	SE Coef	T-Value	P-Value	VIF
Constant	695.2	20.9	33.31	0.000	
G	27.8	16.6	1.67	0.114	1.00
T	34.1	16.7	2.05	0.058	1.00
C	27.8	16.6	1.67	0.114	1.00
E	38.9	16.6	2.34	0.032	1.00
G*G	− 56.5	43.9	− 1.29	0.217	2.93
T*T	100.7	45.4	2.22	0.042	3.10
C*C	43.5	43.9	0.99	0.337	2.93
E*E	− 56.5	43.9	− 1.29	0.217	2.93
G*T	− 37.5	17.6	− 2.13	0.049	1.00
G*C	25.0	17.6	1.42	0.175	1.00
G*E	12.5	17.6	0.71	0.488	1.00
T*C	12.5	17.6	0.71	0.488	1.00
T*E	0.0	17.6	0.00	1.000	1.00
C*E	− 37.5	17.6	− 2.13	0.049	1.00

## Results

In this paper, Eq. (11) is used to determine the optimal problem formulation of each component that is in Full Quadratic mode, and the obtained formulations are reported as follows:

$$Spv = 3967095 - 109 G - 164 T - 27570 C + 42095 E - 0.0226 G*G + 0.294 T*T + 43.5 C*C- 226 E*E - 0.0405 G*T + 0.500 G*C + 0.500 G*E + 0.676 T*C + 0.00 T*E - 75.0 C*E$$

$$Sbat = 112936 + 34.7 G - 223 T - 1680 C + 1873 E - 0.0076 G*G + 0.017 T*T + 5.9 C*C + 24 E*E + 0.0034 G*T - 0.063 G*C + 0.125 G*E + 0.845 T*C + 1.01 T*E - 18.8 C*E$$

$$Sinv = 1096908 + 50.9 G - 51.3 T - 4482 C - 1057 E + 0.00181 G*G - 0.0091 T*T + 4.53 C*C- 9.99 E*E + 0.00203 G*T - 0.1125 G*C - 0.0250 G*E + 0.101 T*C - 0.068 T*E + 3.75 C*E$$

$$Srhp = -813620 + 1.7 G - 30.2 T + 5529 C - 888 E + 0.00242 G*G - 0.0042 T*T - 8.95 C*C+ 24.2 E*E + 0.00159 G*T - 0.0269 G*C - 0.0537 G*E + 0.0726 T*C + 0.145 T*E - 2.94 C*E$$

$$Shs = -78717 + 23.0 G - 59.6 T + 248 C + 2844 E - 0.00379 G*G + 0.1184 T*T - 9.5 C*C- 37.9 E*E - 0.0203 G*T + 0.000 G*C - 0.250 G*E - 0.338 T*C + 2.70 T*E + 12.5 C*E$$

$$Scs = -211787 + 133 G + 102 T - 12666 C + 11608 E - 0.0470 G*G - 0.120 T*T + 160.6 C*C- 190 E*E + 0.0095 G*T + 0.875 G*C + 0.35 G*E + 3.31 T*C - 4.73 T*E + 122.5 C*E$$

$$Sice = 11236044 - 227 G - 159 T - 21458 C - 20708 E + 128165 F + 0.00646 G*G + 0.029 T*T+ 9.9 C*C - 200 E*E + 989 F*F + 0.0203 G*T + 0.225 G*C - 0.450 G*E - 2.25 G*F+ 0.203 T*C - 2.03 T*E - 2.03 T*F + 37.5 C*E - 113 C*F - 375 E*F$$

$$Sbl = -23452 + 14.9 G - 0.0 T - 514 C + 1230 E - 75 F - 0.00292 G*G - 0.0068 T*T + 7.69 C*C- 9.2 E*E - 231 F*F - 0.00541 G*T - 0.0000 G*C - 0.1000 G*E - 0.500 G*F + 0.068 T*C+ 0.135 T*E + 0.68 T*F - 7.50 C*E + 12.5 C*F + 25.0 E*F$$

$$Spat = 55390 - 2.23 G - 1.81 T - 514 C + 311 E - 115.4 H - 0.000016 G*G - 0.00304 T*T+ 1.102 C*C - 4.16 E*E + 0.960 H*H + 0.000203 G*T + 0.00875 G*C + 0.00750 G*E+ 0.00125 G*H + 0.00338 T*C + 0.0068 T*E + 0.01014 T*H + 0.125 C*E + 0.188 C*H- 0.625 E*H$$

$$Sur = 59949 + 6.4 G + 14.4 T + 1360 C - 7429 E + 374 H - 0.00314 G*G - 0.0010 T*T- 7.86 C*C+ 88.6 E*E - 7.86 H*H + 0.00203 G*T + 0.0375 G*C - 0.075 G*E + 0.0750 G*H - 0.101 T*C - 0.203 T*E - 0.000 T*H + 3.75 C*E + 0.00 C*H + 7.50 E*H$$

$$m Fuel = 83595 - 193 T - 116777 C - 0.457 T*T + 41022 C*C + 153.1 T*C$$

**Fig. 3:**
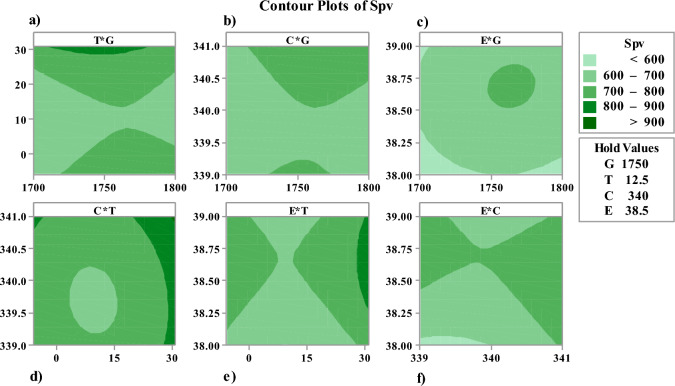
Contour plots of Spv in interaction with (**a**) T*G, (**b**) C*G, (**c**) E*G, (**d**) C*T, (**e**) E*T, (**f**) E*C.

**Fig. 4:**
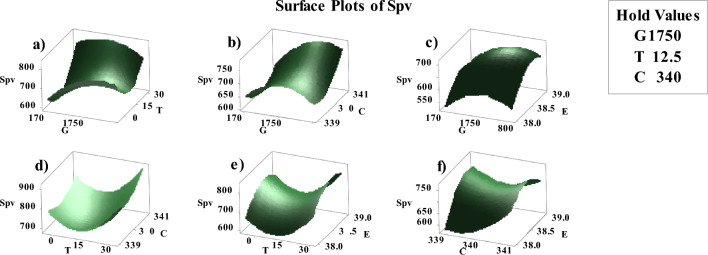
Surface plots of Spv interaction with (**a**) T and G, (**b**) C and G, (**c**) E and G, (**d**) C and T, (**e**) E and T, (**f**) E and C.

The equations presented detail the relationship between various factors (G, T, C, E, F, and H) and the sizing of components $({S}_{pv}, {S}_{bat}, {S}_{inv}, etc.)$. The quadratic nature of these equations highlights both linear and nonlinear interactions. This indicates that the system's performance and component sizing are influenced by both direct and interactive effects of these variables. After evaluating the model and obtaining a mathematical model for each of the problem variables, they can be sensitively analyzed via various plot models. Figures 3 and 4 illustrate the Contour plots and surface plots of ${S}_{PV}$ in interaction with various model parameters, respectively.

Contour Plot is utilized to plot the correlation between the variables of fitted response and two continuous. A contour plot shows 2-dimensional views in which points with the same response values are connected to generate contour lines. Contours can be illustrated by shaded areas, contour lines, or both of them. These graphs help us to show the process of the simulation. Changes from blue to green indicate the improvement in the level of the obtained results. Finally, the higher accuracy in the results is illustrated by darker colors.

**Fig. 5:**
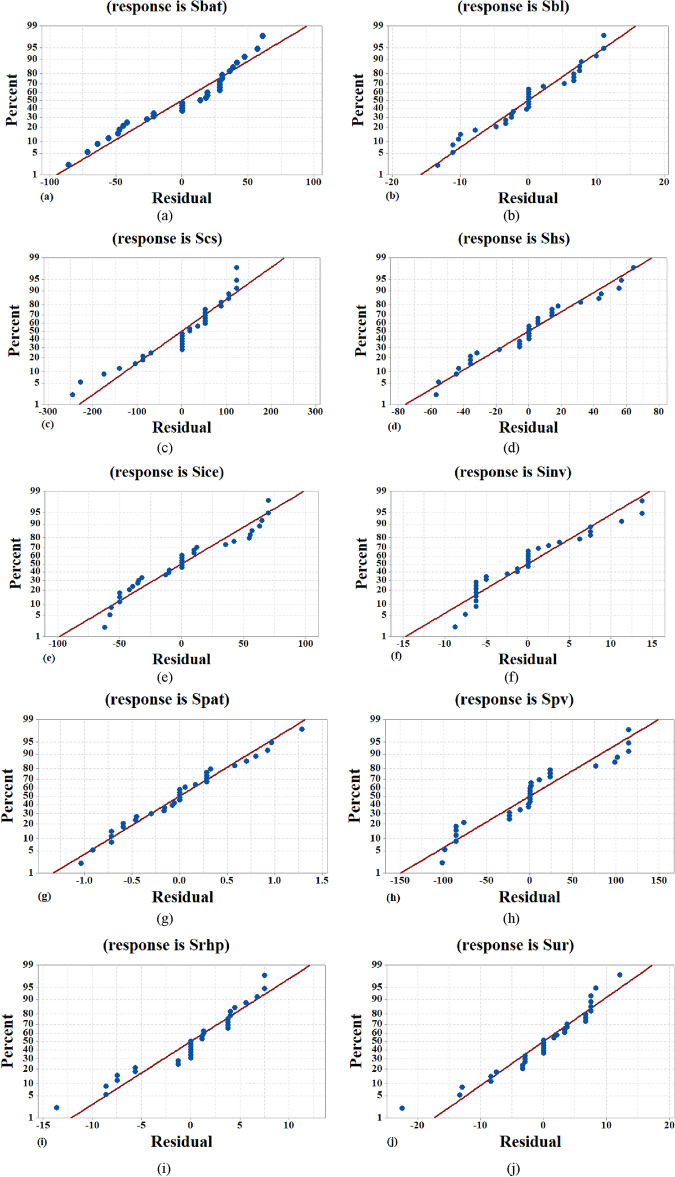
Residual plot related to the response of the various variables; (**a**) ${S}_{bat}$, (**b**) ${S}_{bl}$, (**c**) ${S}_{cs}$, (**d**) ${S}_{hs}$, (**e**) ${S}_{ice}$, (**f**) ${S}_{inv}$, (**g**) ${S}_{pat}$, (**h**) ${S}_{pv}$, (**i**) ${S}_{rhp}$, (**j**)${S}_{ur}$.

Surface plots extract the relationship and correlation between three variables. The variables of predictor are illustrated on two scales of the figure, and the response variable is illustrated on the chart. A contour plot prepares a 2-dimensional view of the surface in which the points with the same response are connected to plot the contour lines that illustrate the constant reactions. Contour plots are advantageous and useful to establish the response values and operating conditions that are desirable. These plots show the order of variables in terms of their effect on ${S}_{PV}$. Figure 5 shows the responses obtained for evaluating each of the variables affecting on the ${S}_{PV}$ in the forms of Residual plot.

The contour and surface plots (Figs. 3 and 4) visually represent the interactions between pairs of variables and their impact on a particular component (e.g., Spv). These plots are crucial for understanding the sensitivity of the component's performance to changes in the variables. For instance, the contour plots show how combinations of temperature and solar irradiance affect the photovoltaic system's size (Spv). The gradients and shapes of these plots help identify regions of optimal operation, where the system's performance is maximized or costs are minimized. This visual analysis supports decision-making by highlighting critical areas where adjustments can lead to significant improvements.

Residual plots are graphs that are tested in the ANOVA environment to check the accuracy of the results. These plots show that the model fits well to optimize all of the independent variables. It is observed that the scatter of data points and standard curves have a high regression coefficient and correlation. Also, the above figures confirm the typical distribution of residuals and the high performance of the models extracted from the studied system.

The residual plots (Fig. 5) validate the model's accuracy in predicting system behavior. The distribution of residuals, which should ideally be random and show no discernible pattern, indicates the goodness of fit of the model. In our study, the residual plots demonstrate a high correlation coefficient, suggesting that the model accurately captures the relationship between the inputs and outputs. This accuracy is crucial for ensuring that the optimization model is reliable and can be used confidently to make decisions about system design and operation. The implications of our findings are multifaceted. Firstly, the detailed equations provide a framework for accurately sizing each component of the hybrid energy system, ensuring that they operate within optimal parameters. This contributes to the system's overall efficiency and cost-effectiveness. Additionally, the sensitivity analysis via contour and surface plots guides system designers in understanding the impact of environmental and economic variables, thus aiding in robust decision-making under varying conditions.

This study relies on data specific to the region of Iran, including climate data, energy consumption patterns, and economic factors. While this data is sufficient for our analysis, we acknowledge that variations in data quality and availability in other regions could impact the applicability and precision of our findings. Future studies should consider the variability and quality of local data to enhance the accuracy and relevance of the model.

The validity of the RSM-based optimization model is contingent upon the assumptions made during the modeling process, such as linearity and the smoothness of the response surface. These assumptions might not hold in all scenarios, particularly in cases involving non-linear interactions or discontinuities in the system behavior. Additional validation with diverse datasets and scenarios would help to verify and refine the model’s accuracy and robustness.

Future research could focus on applying the RSM-based optimization framework to different climates and geographic regions. This would involve collecting and integrating local data, which could provide insights into the model's adaptability and the scalability of the proposed solutions.

Integrating more advanced data analytics techniques, such as machine learning, could improve the accuracy of the response surfaces, particularly in handling non-linearities and complex interactions within the system. This approach could lead to more precise optimization outcomes.

## Conclusion

Today, HESs are used extensively in different areas of the world to supply energy demand. The optimal design of these systems is an important issue and has created many challenges. In this paper, the Response Surface Methodology (RSM) is proposed as a powerful tool for optimal sizing of a Photovoltaic (PV) system in a hybrid energy system (HES). The introduced solution takes into account the climatic and geographical factors in the study site and technical and economic issues related to the HESs, and provides the most optimal sizing related to the PV system. In addition, the proposed technique mathematically modeled each of the variables affecting the performance of the PV system so that the impact of each on the output of the system could be analyzed. Finally, by presenting mathematical models for each input parameter and sensitivity analysis of each of them, the optimal size of the PV system was provided. The optimization model obtained using the analysis of variance (ANOVA) evaluation technique, one of the most important statistical evaluation procedures, was evaluated. It should be noted that the selected RSM model can be considered to optimize all components of a HES.

## Data Availability

The datasets generated and/or analysed during the current study are not publicly available due to extraction from an intra-university project but are available from the corresponding author of dataset on reasonable request.
